# Mitochondria and NMDA Receptor-Dependent Toxicity of Berberine Sensitizes Neurons to Glutamate and Rotenone Injury

**DOI:** 10.1371/journal.pone.0107129

**Published:** 2014-09-05

**Authors:** Kai Kysenius, Cecilia A. Brunello, Henri J. Huttunen

**Affiliations:** Neuroscience Center, University of Helsinki, Helsinki, Finland; University of Texas Medical Branch, United States of America

## Abstract

The global incidence of metabolic and age-related diseases, including type 2 diabetes and Alzheimer's disease, is on the rise. In addition to traditional pharmacotherapy, drug candidates from complementary and alternative medicine are actively being pursued for further drug development. Berberine, a nutraceutical traditionally used as an antibiotic, has recently been proposed to act as a multi-target protective agent against type 2 diabetes, dyslipidemias, ischemic brain injury and neurodegenerative diseases, such as Parkinson's and Alzheimer's disease. However, the safety profile of berberine remains controversial, as isolated reports suggest risks with acute toxicity, bradycardia and exacerbation of neurodegeneration. We report that low micromolar berberine causes rapid mitochondria-dependent toxicity in primary neurons characterized by mitochondrial swelling, increased oxidative stress, decreased mitochondrial membrane potential and depletion of ATP content. Berberine does not induce caspase-3 activation and the resulting neurotoxicity remains unaffected by pan-caspase inhibitor treatment. Interestingly, inhibition of NMDA receptors by memantine and MK-801 completely blocked berberine-induced neurotoxicity. Additionally, subtoxic nanomolar concentrations of berberine were sufficient to sensitize neurons to glutamate excitotoxicity and rotenone injury. Our study highlights the need for further safety assessment of berberine, especially due to its tendency to accumulate in the CNS and the risk of potential neurotoxicity as a consequence of increasing bioavailability of berberine.

## Introduction

The number of patients with diabetes has more than doubled in three decades, and is currently estimated to be nearly 350 million [1]. Management of hyperglycemia is a key intervention strategy in diabetes. In addition to lifestyle-directed interventions, a variety of oral pharmacological agents that improve insulin sensitivity are used to treat type 2 diabetes (T2D) and the prediabetic state [2]. Similarly to diabetes, the prevalence of age-related neurodegenerative diseases, such as Alzheimer's disease (AD), is on the rise, affecting more than 34 million people worldwide [3]. For AD, only symptomatic pharmacotherapies are available. In addition to the industrial drug development efforts for T2D, AD and their prodromal phases, complementary and alternative medicine treatments are actively being explored.

The multifunctional natural compound berberine (BBR), a nutraceutical used in traditional Chinese and native American medicine for centuries, is the principal active component of barberry, goldenseal and other root extracts [4]. Recently, the pharmacological and bioactive properties of BBR have been extensively studied in a variety of models, both *in vitro* and *in vivo*, testing its potency against numerous indications, including dyslipidemias [5], ischemia [6], diabetes [5], arrhythmias [7], cancer [8], Parkinson's disease, and Alzheimer's disease [9], [10]. The proposed actions of BBR in metabolic disorders include insulin receptor upregulation, mitochondrial complex I inhibition, AMP-activated protein kinase (AMPK) activation, low-density lipoprotein (LDL) receptor upregulation, and proprotein convertase subtilisin kexin 9 (PCSK9) downregulation ([11], reviewed in [12]). Additionally, cholinesterase inhibition, monoamine oxidase inhibition, BACE1 inhibition and antioxidant activity comprise the proposed anti-AD effects of BBR [13]–[15]. Although numerous mechanisms have been proposed, the pharmacological actions of BBR remain incompletely understood, especially in the central nervous system (CNS).

The lack of clarity within BBR literature is partially due to the variability in the concentrations and formulations of BBR applied in both cell- and animal model-based studies. The common dosage range spans from 0.1 nM to 300 μM, and from 5 mg to 100 mg/kg/day in *in vitro* and *in vivo* studies, respectively [13], [16]–[19]. Pharmacological data suggest that berberine has poor bioavailability and that only nanomolar plasma concentrations are reached in both humans and animals [20]. According to several reports, however, BBR accumulates in organs such as lungs, liver and the brain, resulting in effective concentrations in the low micromolar range [13], [20]–[22]. Additionally, pro-drug development efforts aim at increasing BBR bioavailability [11], [23]. BBR is generally considered safe for use in humans, but several reports have raised concerns over BBR toxicity and side effects, especially with increasing BBR concentrations [19], [21], [24]–[26].

The proposed anti- and pro-apoptotic roles of BBR are largely dose-dependent. In general, nanomolar BBR is believed to protect neuronal cells from ischemic insults, whereas cancer cell growth and proliferation is inhibited by high micromolar BBR [8], [16]. Similarly, in the TgCRND8 mouse model of AD, a higher dosage (100 mg/kg/d versus 25 mg/kg/d) blunted the protective effects of BBR against amyloid plaque pathology and gliosis [13]. The mechanistic basis for the biphasic effects of BBR on neuronal viability remains incompletely characterized [27]. The proposed targets of BBR associated with neuronal viability include modulation of mitochondrial and caspase pathways, N-methyl-D-aspartate (NMDA) receptors, inhibition of potassium currents, and transcriptional regulation of lipoprotein receptors [20].

The characterization of BBR effects on neuronal viability remains incomplete. In this study, we assessed the effect of BBR on neuronal viability using cultured primary neurons: cerebellar granule neurons (CGN) and rat hippocampal neurons (HCN). We found that concentrations exceeding 1 μM reduced neuronal viability in a caspase-independent manner characterized by early alterations of mitochondrial function and morphology. Cyclosporine A (CsA), a mitochondrial permeability transition pore (PTP) inhibitor, could partially prevent BBR toxicity independently of its calcineurin inhibitor activity. However, NMDA receptor antagonists MK-801 and memantine completely blocked this acute toxicity, indicating a central role for NMDA receptors in BBR-mediated cell death. Additionally, subtoxic nanomolar BBR pretreatment sensitized neurons to both glutamate excitotoxicity and rotenone-induced cell death. Our study provides mechanistic evidence of a neurotoxic mechanism of BBR that involves the NMDA receptors and mitochondria, predisposing neurons to glutamate excitotoxicity and mitochondria-targeting toxins.

## Materials and Methods

### Ethics statement

Animals were obtained from the Laboratory Animal Center, University of Helsinki. Animals were housed in controlled conditions (temperature +22°C, light from 08∶00 to 18∶00; humidity 50–60%), with fresh food and water available *ad libitum*. Tissue extraction from NMRI mice and Wistar rats was performed in accordance with the national and institutional guidelines approved by the University of Helsinki Laboratory Animal Center (permission number KEK11-019; approved 31.5.2011). Animals were euthanized by approved methods (CO_2_ chamber and cervical dislocation) prior to the extraction of brain tissue used for primary neuronal culture preparation. A total of 65 P6–8 NMRI mouse pups and 4 Wistar pregnant rats and their litters were sacrificed for the generation of the primary cell cultures used to generate the data presented in this study.

### Reagents

Berberine chloride, z-VAD-FMK, cyclosporine A, memantine, MK-801, and resazurin sodium salt were purchased from Sigma. FK506 (cat. no. Asc-223) was purchased from Ascent Technologies. Rotenone was a kind gift from Dr. Timo Myöhänen (Faculty of Pharmacy, University of Helsinki). CellTiter-Glo Luminescent Cell Viability Assay (cat. no. G7570) and CytoTox 96 Non-Radioactive Cytotoxicity Assay (cat. no. G1780) kits were purchased from Promega. JC-10 Mitochondrial Potential Assay Kit (cat. no. ab112123) was purchased from Abcam. CM-H_2_DCFDA General Oxidative Stress Indicator (cat. no. C6827) was purchased from Life Technologies.

### Molecular cloning

The GFP-tagged mitochondrial outer membrane protein-25 (GFP-OMP25) plasmid [28] was a kind gift from Dr. Brendan Battersby (Faculty of Medicine, University of Helsinki). The GFP-OMP25 fragment was cloned into the lentiviral expression vector with synapsin (pLenSyn1) promoter for specific transduction into neurons [29]. The GFP-OMP25 cassette was amplified by PCR (primers used: 5′-CGGGATCCGCCACCATGGTGAGCAA-3′and 5′-CGCGCTCGCGCTATTAGAGCTGCTTTC-3′). Both the PCR fragment and the vector were digested with the restriction enzymes BamHI and XhoI. Following the transformation of XL1 Blue *E. coli* with the new ligated plasmid, colonies were screened by NheI digestion and confirmed by sequencing.

### Lentivirus production

Lentiviral particles were produced as described previously [30]. Briefly, HEK-293T cells were cultured in DMEM (supplemented with 10% FBS, 1% Penicillin/Streptomycin and 2 mM L-glutamine) and grown on 10-cm plates to 60–80% confluency. The cells were transfected using Fugene HD (Promega) with 6 µg of total DNA per plate, including pLenSyn1-GFP-OMP25 (3 µg) and the viral envelope and packaging plasmids pMD2.G (0.75 µg) and pPAX2 (2.25 µg), respectively. The supernatant was collected 45 hours post-transfection and precleared by centrifugation at 900 rpm 3 times for 5 minutes. Viral particles were collected by centrifugation at 50,000×g for 2 hours, resuspended in plain DMEM and stored at −80°C prior to use.

### Preparation of primary neurons and lentiviral transduction

Cerebellar granule neurons (CGN) and hippocampal neurons (HCN) were prepared as described previously [30]. Briefly, CGN were prepared from P6–P8 NMRI mice and HCN from E18 Wistar rat embryos. Brain tissues were dissected and cleaned of membranes in cold PBS supplemented with 0.25% glucose, 0.3% bovine serum albumin (BSA), and 0.038% MgSO_4_. Tissues were trypsinized for 15 minutes in a +37°C water bath. CGN were plated on poly-L-lysine (Sigma) coated cell culture plates at a density of 0.325–0.5 million cells per ml and HCN at a density of 0.15–0.2 million cells per ml. HCN were grown in Neurobasal (Gibco) supplemented with 2% B27, 2 mM L-glutamine, and 1% Penicillin/Streptomycin. CGN culture medium included additional 0.5% FBS and 25 mM potassium chloride, required for sustaining CGN cultures *in vitro*. Neurons were cultured for 6–7 days before the start of treatments. CGN were transduced at 4 DIV and cultured for 72–96 hours prior to the start of treatments.

### Treatments

BBR was prepared freshly before each set of experiments by dissolving in DMSO as a 20 mM stock solution. Culture concentrations (0.01–10 µM) were made by serial dilutions into plain Neurobasal medium with total DMSO content remaining below 0.1%. Neurons were treated with BBR for 0.5–24 hours, as indicated in the data. For cotreatment experiments, neurons were pretreated with z-VAD-FMK (100 µM), FK-506 (1 µM), CsA (1 µM), memantine (10 µM), and MK-801 (1 µM) for 1 hour before the addition of BBR for additional 6 hours. For BBR preconditioning experiments, CGN were pretreated with 30 or 300 nM BBR for 18 hours before the addition of glutamate (20–100 µM) or rotenone (0.1–1 µM) into the culture medium for 6 hours. CGN were deprived by switching to low potassium/serum medium for 6 hours (K5) to induce neuronal apoptosis and caspase-3 activation (Neurobasal supplemented with 0.5% FBS, 1% Penicillin/Streptomycin, 2 mM L-glutamine and 5 mM KCl).

### CellTiter-Glo cell viability assay

ATP-based CellTiter cell viability was measured according to the manufacturer's instructions (Promega). Briefly, CGN were plated on 96-well white-walled clear-bottom plates and grown for 7 DIV. After completion of experiments, 100 µl of pre-mixed CellTiter-substrate was added to each well and the plate was shaken for 2 minutes at 200 rpm. The luminescence was then measured (Ex/Em  = 560/590 nm) with the Victor^3^ 1420 Multilabel counter.

### Immunofluorescence microscopy

Immunofluorescence (IF) microscopy was performed as previously described [30]. Briefly, cells were grown on poly-L-lysine coated coverslips and fixed for 20 minutes with 4% PFA in PBS before permeabilization for 1 hour in blocking buffer (5% normal serum [goat and donkey], 1% BSA, 0.1% gelatin, 0.1% Triton-X, 0.05% Tween-20). Primary antibodies used were: β-tubulin III (TUJ1; Covance, mouse and rabbit, 1∶1000), TOM-20 (a kind gift from Prof. Anu Wartiovaara [Faculty of Medicine, University of Helsinki]; Santa Cruz, rabbit, 1∶1000), and AIF (D39D2; Cell Signaling, rabbit, 1∶500). Primary antibodies were incubated overnight at +4°C. The secondary antibodies used were: AlexaFluor-conjugated antibodies (Invitrogen) 488-goat-anti-mouse, 488-donkey-anti-rabbit, 350-goat-anti-mouse, and 568-donkey-anti-rabbit, used at a dilution of 1∶2000. Secondary antibodies were incubated for 1 hour at room temperature. Cell nuclei were stained with Hoechst 33342 (Invitrogen, 1∶10,000). Images were taken with a Zeiss Imager M1 microscope. ImageJ software was used for cell counting and Adobe Photoshop for the preparation of figures.

### Neuronal cell viability

Neuronal cell viability was evaluated by assessing nuclear morphology from immunofluorescence images taken at 20X and 40X magnifications from random fields. The nuclei of TUJ-1-positive cells, identified as neurons, were counted and scored as either normal or condensed as previously described [30]. Neuronal viability was calculated by the following formula: (total neuronal nuclei – condensed neuronal nuclei)/total neuronal nuclei. At least 300 cells per coverslip were counted for each data point.

### Western blotting

Western blotting (WB) was performed as previously described [30]. Briefly, cells were lysed in extraction buffer containing 10 mM Tris-HCl, pH 7.6, 2 mM EDTA, 0.15 M NaCl, 1% Triton-X, inhibitor cocktail (1 pill/10 ml; Roche), and 0.25% NP-40 (Sigma). Lysates were cleared by centrifugation at 13,000×g for 10 minutes. Samples were denatured with β-mercaptoethanol and heated at 70°C for 10 min before running them in NuPAGE 4–12% Bis-Tris gels (Invitrogen) at 160 V for 60 minutes using the X-Cell SureLock (Invitrogen) gel apparatus. Proteins were transferred to PVDF membranes with a Bio-Rad Trans-Blot Turbo for 25 minutes at 25 V. Membranes were blocked for 1 hour in 5% non-fat milk powder (Valio) diluted in TBST (TBS +0.1% Tween-20 [Sigma]) before overnight incubation with primary antibodies at +4°C. The primary antibodies used were: cleaved caspase-3 (Asp175; Cell Signaling, rabbit, 1∶1000), phospho-c-Jun (Ser65; Cell Signaling, rabbit, 1∶1,000), β-tubulin I+II (Sigma, mouse, 1∶1000), and GAPDH (6C5; Millipore, mouse, 1∶1000). On the next day, membranes were washed 3 times for 10 minutes with TBST and an appropriate secondary antibody was added for 1 hour. Horseradish peroxidase-linked anti-mouse (GE Healthcare, LNA931V) and anti-rabbit (GE Healthcare, LNA934V) secondary antibodies were used at a 1∶6000 dilution in TBST. After washing, membranes were soaked for 3 minutes with Pierce ECL reagent (Thermo Scientific, 32106). Protein bands were detected with the LAS-3000 imaging system (Fujifilm). Quantity One software (Bio-Rad) was used for the optical density quantification of Western blots.

### CM-H_2_DCFDA oxidative stress assay

CM-H_2_DCFDA is a probe sensitive to oxidation by reactive oxygen species (ROS) and was used to measure oxidative stress according to the manufacturer's instructions (Life Technologies). Briefly, CGN were cultured on 96-well white-walled clear-bottom plates in phenol-red free Neurobasal media until 7 DIV. Before the start of experiments, all wells were washed once with prewarmed PBS and the cells were loaded with 100 μl per well of CM-H_2_DCFDA (50 μg diluted in 6 milliliters of PBS) for 30 minutes at 37°C. Fresh phenol-red free Neurobasal media and BBR treatments (BBR 0.01–10 μM) were added to the wells after loading. The fluorescence was measured (Ex/Em  = 485/515 nm) with the Victor^3^ 1420 Multilabel counter after 0.5, 1, 2, 4 and 6 hours post-treatment.

### JC-10 mitochondrial membrane potential assay

Mitochondrial membrane potential was measured according to the manufacturer's instructions (Abcam). Briefly, CGN were cultured on 96-well white-walled clear-bottom plates in phenol-red free Neurobasal until 7 DIV. Thirty minutes before the end of the treatment, 50 μl of JC-10 dye-loading solution was added to each well and incubated for 30 minutes before measuring fluorescence intensities (Ex/Em  = 485/515 nm and Ex/Em  = 540/590 nm). The change of mitochondrial membrane potential was measured as the ratio between aggregate (Em  = 515 nm) and monomeric forms (Em  = 590 nm) of JC-10. Increasing ratios indicate mitochondrial membrane depolarization.

### Mitochondrial metabolic activity assay

The rate of mitochondrial metabolic activity was assessed with the resazurin reduction assay [31]. Briefly, CGN were cultured on 96-well white-walled clear-bottom plates until 7 DIV. Resazurin was added to each well to yield a final concentration of 0.1 mM. BBR and rotenone treatments were added to appropriate wells and the fluorescence intensity of reduced resazurin (Ex/Em  = 540/590 nm) was measured hourly with the Victor^3^ 1420 Multilabel counter for 6 hours, with the maximal reduction (100%) measured after 24 hours from vehicle-treated control wells. Rotenone was used to block complex I activity in order to determine the resazurin reduction rate independent of complex I activity.

### LDH cytotoxicity assay

LDH measurements were performed as described in the manufacturer's instructions (Promega). Briefly, 50 µl of conditioned media from CGN cultures were transferred into 96-well white-walled clear-bottom plates (PerkinElmer) in duplicate and 50 µl of LDH substrate was added to each well. The plate was incubated for 30 minutes at room temperature in the dark before the addition of 50 µl stop solution. Freshly prepared culture medium was included as a negative control. After gentle mixing on a plate shaker, absorbance was measured at 490 nm with the Victor^3^ 1420 Multilabel counter (PerkinElmer).

### Statistical analyses

A minimum of three repetitions from at least two different batches of cells were used for each experiment. Microsoft Excel and GraphPad Prism software were used for statistical analyses and generation of graphs. Statistical significance was evaluated with the Student's t-test and ANOVA, with the significance threshold set at p<0.05 (*).

## Results

### Dose-dependent effects of berberine on the viability of primary neurons

**Figure 1:**
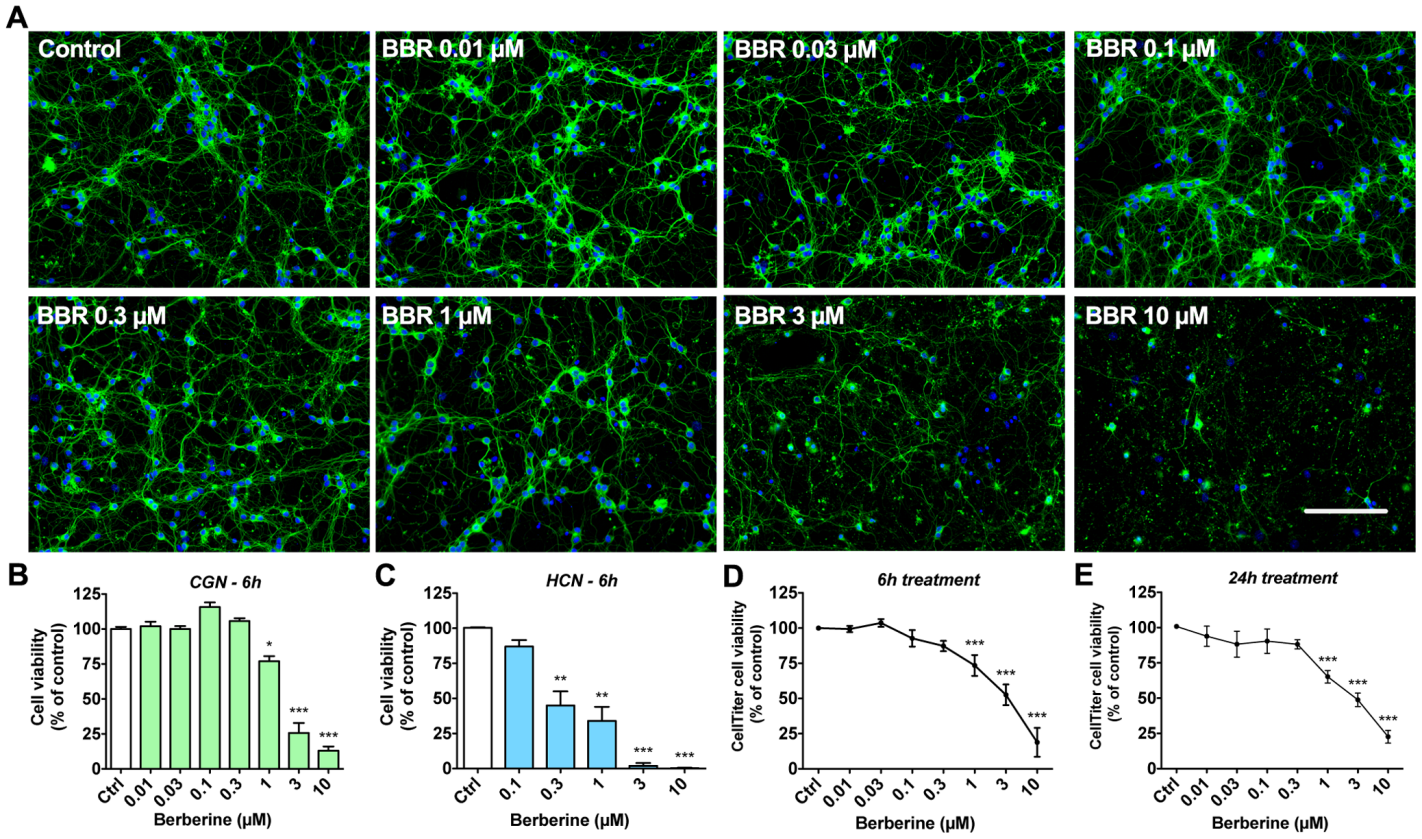
Dose-dependent neurotoxicity of berberine in primary neurons. Primary neurons (cerebellar granule neurons [CGN; DIV7] and hippocampal neurons [HCN; DIV7]) treated with BBR for 6 hours at concentrations between 0.01 and 10 µM indicate a clear dose-dependent loss of neuronal cell viability with IC_50_ of roughly 3 µM. (A) CGN were stained for visualization of neurite (β-tubulin III, TUJ1) and nuclear (Hoechst 33342) morphology at 20X magnification. (B) Neuronal cell viability assessed by visual scoring of CGN nuclear morphology after 6-hour treatment with BBR. (C) Neuronal cell viability assessed by visual scoring of HCN nuclei morphology after 6-hour treatment with BBR. (D) Neuronal viability of CGN, as assessed by CellTiter-Glo assay measuring cellular ATP content, for 6-hour treatments with the indicated BBR concentrations. (E) Neuronal viability of CGN, as assessed by CellTiter-Glo assay for 24-hour treatments with the indicated concentrations. For A, green is β-tubulin III, blue is Hoechst; the scale bar represents 100 µm. For panels B, D, E, n = 5. For C, n = 3. For B–E, *  = p<0.05, **  = p<0.01, ***  = p<0.001.

We evaluated the effects of BBR on primary neuron viability by immunofluorescence (IF)-based morphological analysis and the CellTiter-Glo ATP-based cell viability assay. We treated CGN with BBR concentrations ranging from 10 nM to 10 µM for 6 hours and assessed neuronal viability by visualizing nuclear morphology with Hoechst staining. As shown in Figure 1A, lower (0.3 µM and under) concentrations of BBR did not significantly affect the gross morphology of the CGN neuritic network, comprising both neuronal dendrites and axons. Treatment with 0.1 µM BBR caused a modest increase in cell viability determined by nuclear staining (Figure 1B) but not ATP content (Figure 1D). However, concentrations exceeding 1 µM caused a severe disruption of neuritic and nuclear integrity (Figure 1A). These concentrations reduced cell numbers and increased the percentage of condensed nuclei observed in culture (Figure 1B). In HCN, BBR treatments yielded a similar reduction of viability (Figure 1C), indicating a general neurotoxic property for BBR.

Additionally, we assessed cell viability by measuring the ATP content of the neuronal cultures. Treatments with BBR (0.01–10 µM) showed a similar decrease in ATP levels after both 6- and 24-hour treatments (Figure 1D, E). Similar to cell viability assessment by nuclear morphology, BBR significantly reduced the neuronal ATP content at concentrations between 1 to 10 µM, with an IC_50_ of ≈3 µM for both 6- and 24-hour treatment periods. Ten micromolar BBR caused the largest decrease, 81.2±10.2% in ATP levels (Figure 1D), and a 87.0±3.0% (Figure 1B) decrease in neuronal cell viability, and a dramatic disruption of neuritic integrity (Figure 1A). Consequently, 10 µM BBR was used as the most potent toxic dose in further experiments.

### Berberine causes functional and morphological alterations of neuronal mitochondria independent of caspase-3 activation

**Figure 2:**
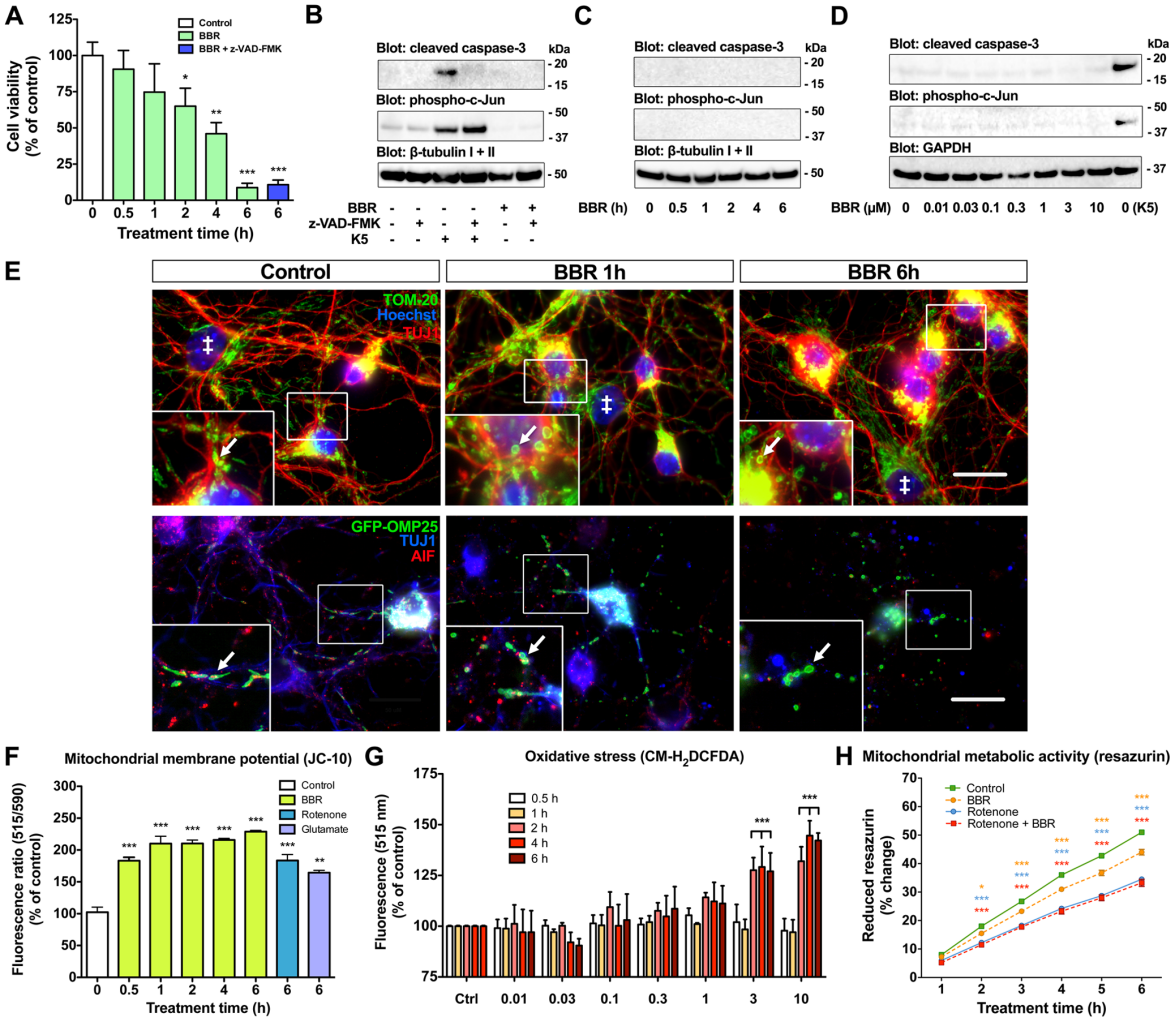
Berberine alters mitochondrial function and morphology, and causes caspase-independent cell death. BBR causes rapid reduction in cell viability independent of caspase-3 activation, and changes in mitochondrial function and morphology. (A) Neuronal cell viability assessed by visual scoring of nuclear morphology reveals the largest drop in viability to take place between 4 and 6 hours. Pan-caspase inhibitor z-VAD-FMK does not affect BBR toxicity after cotreatment for 6 hours. (B) Western blot (WB) of CGN cell lysates shows caspase-3 cleavage and phosphorylation of c-Jun in serum/potassium deprived-neurons (K5) but not in BBR-treated neurons. Cotreatment with z-VAD-FMK prevents caspase-3 cleavage, but c-Jun remains phosphorylated as it functions upstream of caspase-3 in the neuronal apoptosis pathway. (C, D) WB of CGN lysates show that treatment with 10 µM BBR for 0.5–6 hours (C) or with 0.01–10 µM BBR for 6 hours (D) does not induce cleavage of caspase-3 or phosphorylation of c-Jun. (E) CGN were stained with TOM-20 (top row) or transduced with GFP-OMP25 lentivirus (bottom row) to visualize all mitochondria and neuronal mitochondria, respectively. Glial cells, marked with ‡, display larger nuclei, and lack of TUJ1-staining (top row). Mitochondria in untreated neurons display an elongated shape, and the proportion of rounded mitochondria (white arrows) is increased by BBR treatment. (F) BBR lowers mitochondrial membrane potential in CGN as evaluated by the increased JC-10 monomer/aggregate ratio (515/590 nm). (G) BBR at 3 and 10 µM causes a sharp increase in oxidative stress after 2 hours of treatment as assessed by the CM-H_2_DCFDA assay. (H) BBR treatment lowers the rate of resazurin reduction, but does not have an additive effect when coupled with maximal complex I inhibition with 10 μM rotenone. Orange *** indicate significant difference between control and BBR, blue *** indicate significant difference between control and rotenone, and red *** between BBR and BBR + rotenone-treated CGN. For E: (top row) blue is Hoechst, green is TOM-20 and red is β-tubulin III and (bottom row) blue is β-tubulin III, green is GFP-OMP25 and red is AIF; the scale bar represents 20 µm. For panels A, F, H, n = 4. For panel G, n = 5. For A, F, G and H, *  = p<0.05, **  = p<0.01, ***  = p<0.001.

BBR triggers both caspase-dependent and -independent pathways of apoptosis [32]–[34]. Hence, we assessed the time frame of cell death, and the role of caspases and mitochondria to elucidate the mechanisms of BBR neurotoxicity. In a time interval trial, 10 µM BBR steadily reduced CGN viability, with the most robust loss of viability taking place between 4 and 6 hours of treatment, dropping from 46.0±7.6% to 9.0±2.3% (Figure 2A). Additionally, this loss of viability was insensitive to pan-caspase inhibitor z-VAD-FMK (100 µM), indicating a caspase-independent pathway (Figure 2A). We verified the efficacy of z-VAD-FMK caspase inhibition from cell lysates by WB (Figure 2B). The induction of caspase-3 cleavage, the main executioner caspase in neurons, was blocked by z-VAD-FMK during neuronal apoptosis caused by serum/potassium deprivation (K5) leaving the upstream c-Jun phosphorylation unaffected, whereas CGN treated with 10 µM BBR for 6 hours showed no phosphorylation of c-Jun or caspase-3 cleavage (Figure 2B). Additionally, we detected no caspase-3 cleavage or phosphorylation of c-Jun in CGN treated with 10 µM BBR between 0.5–6 hours (Figure 2C) or with BBR concentrations 0.01–10 µM (Figure 2D). These data indicate that BBR-induced neuronal cell death is a rapid caspase-independent process.

Mitochondria are a known target of BBR and a central player in multiple cell death pathways [35]. We assessed the morphological changes of mitochondria by visualizing outer mitochondrial membrane protein TOM-20 by IF-staining (Figure 2E; top row). To better selectively visualize neuronal mitochondria, we transduced CGN with synapsin-promoter driven expression of GFP-OMP25 [28], [29] coupled with IF-staining (Figure 2E; bottom row). BBR treatment induced mitochondrial morphological changes in TUJ1-positive cells resembling swelling already after one hour (white arrows in Figure 2E). In contrast to control cells, BBR increased the proportion of spherical mitochondria (Figure 2E). These morphological changes indicate that BBR induces mitochondrial swelling, known to associate with the opening of the mitochondrial permeability transition pore (PTP), a drop in the mitochondrial membrane potential and loss of mitochondrial Ca^2+^ retention [22], [35].

In order to elucidate a possible mechanism for mitochondrial swelling, we further explored the effects of BBR on the mitochondrial membrane potential and oxidative stress. Mitochondrial membrane potential was assessed by fluorescent JC-10 that selectively enters the mitochondria forming reversible aggregates as the mitochondrial membrane becomes more polarized, shifting the emitted light from 515 nm (monomeric form) to 590 nm (aggregate form). The mitochondrial membrane potential in CGN treated with 10 µM BBR dropped already after 30 minutes as indicated by the increase in monomer/aggregate ratio (Figure 2F). Treatments with 10 µM rotenone and 100 µM glutamate show a similar loss of membrane potential after 6 hours (Figure 2F). In contrast, oxidative stress increased in neurons treated with 3 and 10 µM BBR only after 2 hours (Figure 2G), coinciding with a decrease in viability seen in Figure 2A. Lastly, the effect of BBR on mitochondrial metabolism was assessed by resazurin reduction assay. BBR lowered the rate of resazurin reduction during 2 to 6 hours of treatment (yellow *) from 18.0±0.4% to 15.5±0.5% at 2 hours, and from 51.0±0.6% to 44.0±1.0% at 6 hours (Figure 2H). Full inhibition of mitochondrial complex I with 10 μM rotenone significantly reduced the rate of resazurin reduction but was not further lowered by BBR treatment (Figure 2H) suggesting that BBR and rotenone do not have an additive effect on mitochondrial metabolism. These data indicate that mitochondrial membrane depolarization and mitochondrial swelling precede an increase in oxidative stress and loss of cell viability in neurons exposed to BBR.

### Cyclosporine A partially protects neurons from BBR toxicity

**Figure 3:**
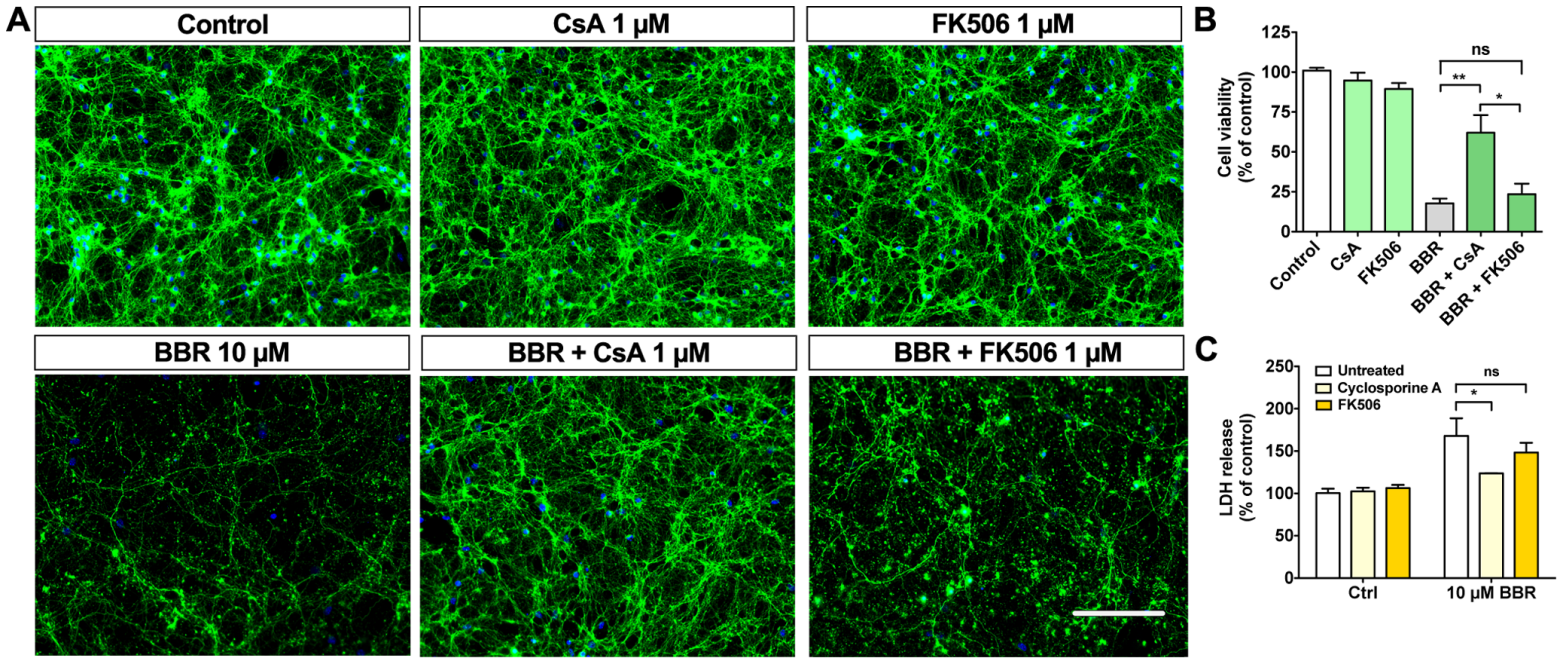
Cyclosporine A partially blocks berberine toxicity. Pretreatment with CsA partially protects CGN against BBR toxicity. (A) Representative images of CGN pretreated 1 hour with and without 1 µM CsA or FK506 before the addition of 10 µM BBR. (B) Quantification of CGN cell viabilities shown in A. (C) LDH release assay from the conditioned media for the described treatments shows partial protection by CsA, but not for FK506. For A, green is β-tubulin III, blue is Hoechst; the scale bar represents 100 µm. For panel B, n = 4 and for C, n = 3. For B and C, *  = p<0.05, **  = p<0.01.

Cyclosporine A (CsA) is a potent PTP inhibitor used as an immunosuppressant and to prevent cardiac hypertrophy following ischemic or reperfusion injuries. Moreover, CsA has been proposed to interact with herbal supplements, such as berberine [36], [37]. We used CsA to elucidate whether BBR-induced neurotoxicity is dependent on the PTP formation. Our data indicate that CsA can partially protect neurons from BBR-induced neurotoxicity (Figure 3A), as cell viability improved from 13.3±2.8% to 63.5±11.4% in the presence of 1 µM CsA as assessed by nuclear morphology (Figure 3B). The release of LDH was also reduced from 168±20.7% to 124±0.3% (Figure 3C). Since CsA also inhibits calcineurin activity, we also assessed the effect of FK506, a specific calcineurin inhibitor that has no effect on PTP, on the viability of BBR-treated CGN. FK506 failed to protect CGN from BBR neurotoxicity (Figure 3B, C). These data further highlight the importance of mitochondria in BBR-mediated toxicity and suggest that PTP formation is involved in mediating the neurotoxic effects of BBR.

### NMDA receptor antagonists memantine and MK-801 prevent berberine toxicity

**Figure 4:**
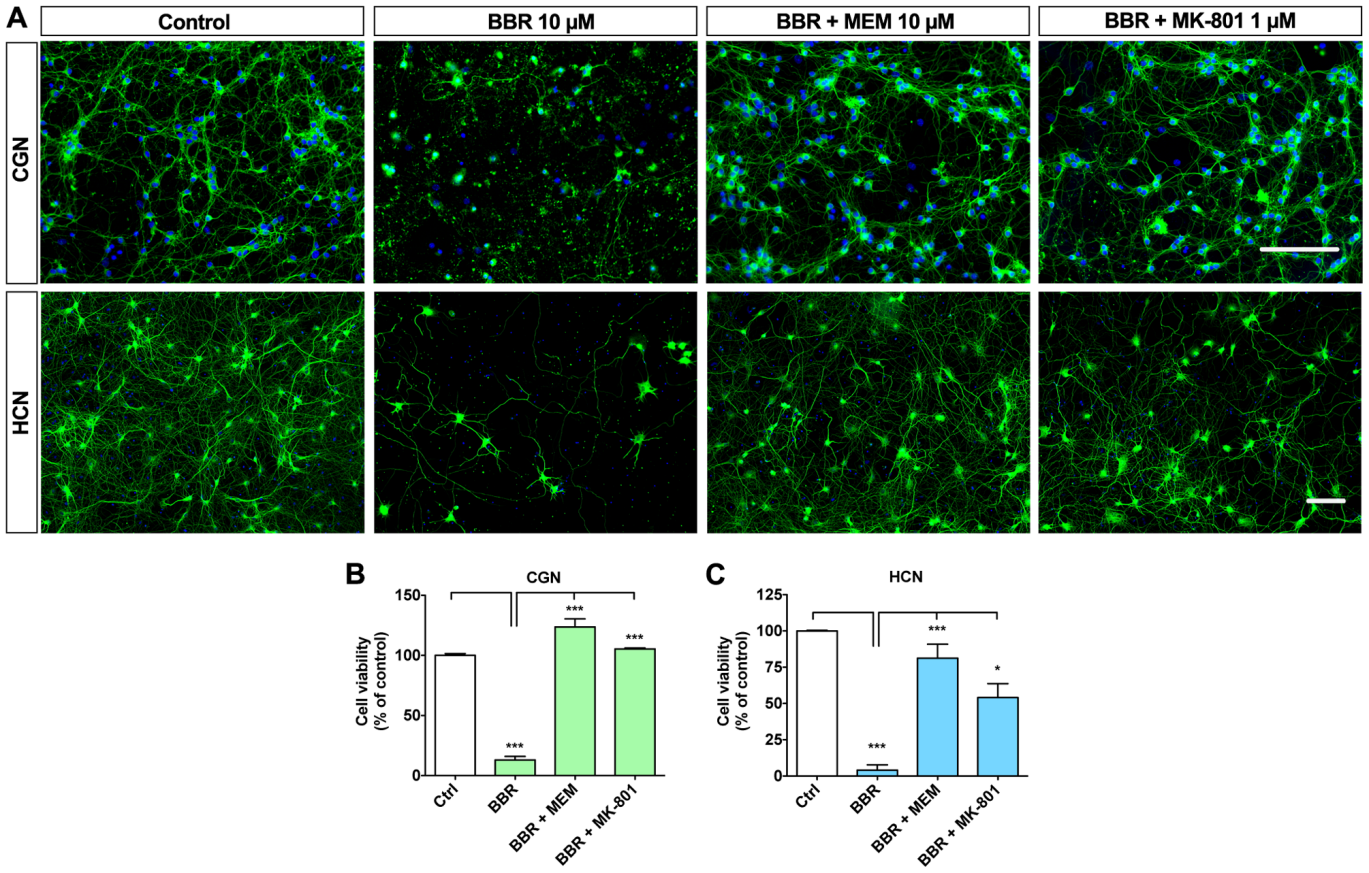
NMDAR antagonists memantine and MK-801 block BBR-induced cell death. NMDA receptor antagonists memantine and MK-801 block toxicity caused by 10 µM BBR in CGN and HCN cultures. (A) Representative IF images of CGN (upper row) and HCN (lower row) pretreated with memantine and MK-801 before the addition of 10 µM BBR for 6 hours. (B) Quantification of CGN viabilities shown in A. (C) Quantification of neuronal cell viability of IF images of HCN. For A, green is β-tubulin III, blue is Hoechst; the scale bars represent 100 µm. For panels B and C, n = 3. For B and C, *  = p<0.05, ***  = p<0.001.

Excitotoxic neuronal cell death is often accompanied by formation of the mitochondrial PTP and release of pro-apoptotic factors from mitochondria to cytosol [38], [39]. NMDA receptors and mitochondria are functionally coupled in many types of neuronal cell death [40]. The time course of BBR-induced cell death is faster than in programmed apoptosis and resembles mixed necroptosis occurring during excitotoxic injury [30], [41], [42]. Glutamate excitotoxicity in neurons is characterized by mitochondrial defects, increased intracellular Ca^2+^ and activation of caspase-independent cell death pathways, and can be prevented by blocking NMDA receptors [43], [44]. To elucidate whether BBR toxicity is dependent on NMDA receptor function, we pretreated CGN with NMDA receptor antagonists MK-801 or memantine for 1 hour before the addition of 10 µM BBR for 6 hours. Both NMDA receptor antagonists preserved neuritic network morphology and completely blocked BBR-induced cell death (Figure 4A, B). Similarly to CGN cultures, NMDA receptor antagonists also prevented BBR toxicity in HCN (Figure 4A, C). These pharmacological data suggest that NMDA receptors are centrally involved in BBR neurotoxicity.

### Berberine pretreatment sensitizes neurons to glutamate excitotoxicity

**Figure 5:**
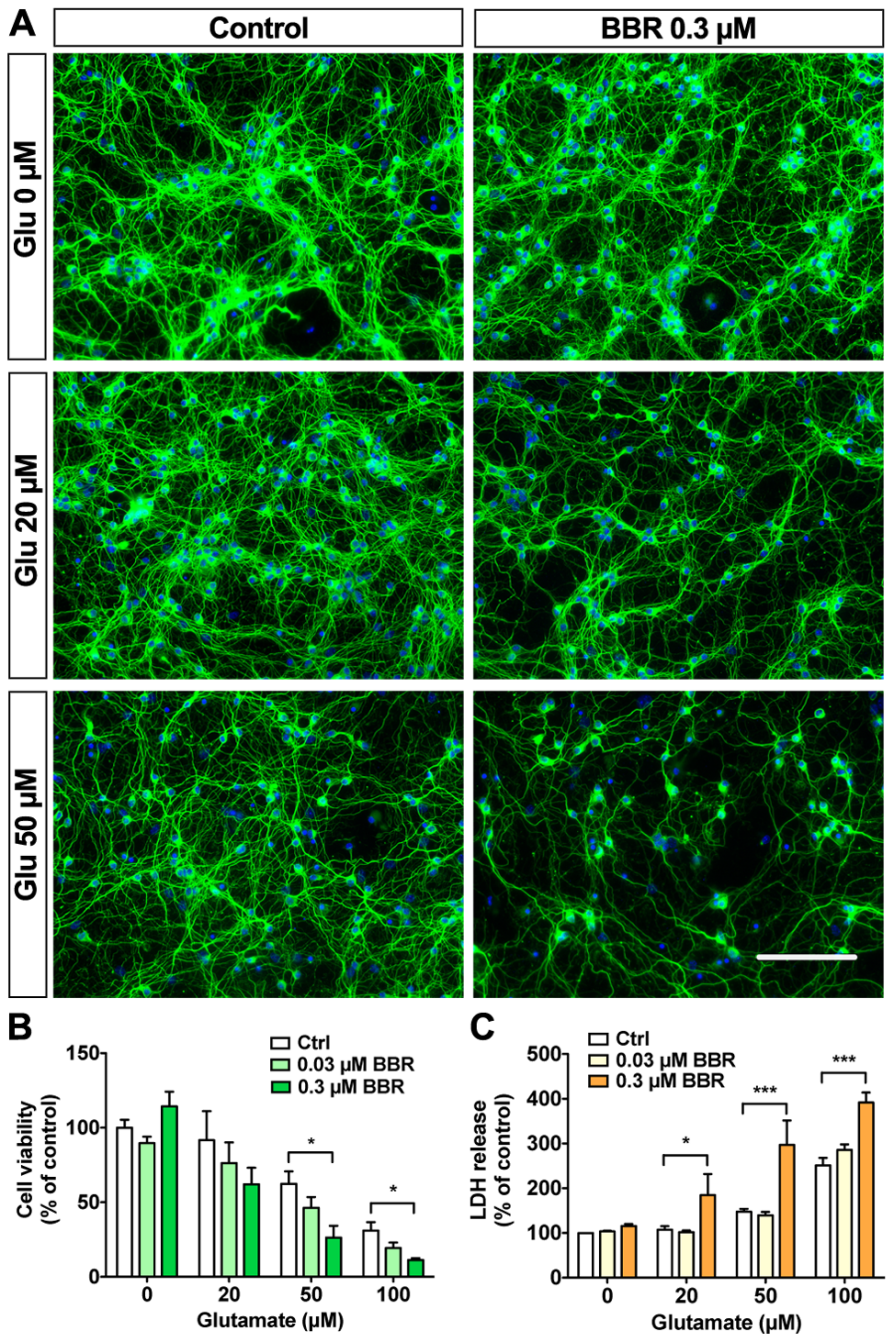
Berberine sensitizes neurons to glutamate toxicity. Pretreating CGN 18 hours with 300 nM BBR exacerbates glutamate excitotoxicity. (A) Representative IF images for CGN pretreated with 300 nM BBR for 18 hours before the addition of 20 or 50 µM glutamate for 6 hours. (B) Quantification of CGN cell viabilities shown in A. (C) LDH release assay from the conditioned media for the described treatments shows exacerbation of toxicity by 300 nM BBR with glutamate co-treatments. For A, green is β-tubulin III, blue is Hoechst; the scale bar represents 100 µm. For panels B, n = 3 and panel C, n = 4. For B and C, *  = p<0.05, ***  = p<0.001.

Although our results suggest that BBR is neurotoxic at low micromolar concentrations, previous research indicates that low nanomolar BBR pretreatment can elicit neuroprotection in neuronal-type cells against hypoxic and ischemic insults [16]. As glutamate excitotoxicity is the primary cause of neuronal cell death in ischemic stroke, we tested whether nanomolar BBR can either sensitize neurons to or protect them from glutamate excitotoxicity. Previous research has determined a subtoxic range of glutamate exposure in CGN between 20–50 µM, whereas 100 µM glutamate produces prominent excitotoxicity within 6 hours [45], [46]. We pretreated CGN for 18 hours with 30 or 300 nM BBR (well-tolerated concentrations; Figure 1) before the addition of 20, 50, or 100 µM glutamate for an additional 6 hours. When pretreated with 300 nM BBR, CGN became more susceptible to glutamate excitotoxicity, as evidenced by neuritic degeneration (Figure 5A), nuclear condensation (Figure 5B), and LDH release (Figure 5C). Although displaying a similar trend of sensitization as 300 nM BBR, pretreatment with 30 nM BBR did not significantly either increase or reduce neuronal viability. These data suggest that subtoxic nanomolar BBR concentrations are sufficient to sensitize neurons to glutamate excitotoxicity [47].

### Nanomolar BBR sensitizes neurons to rotenone injury

**Figure 6:**
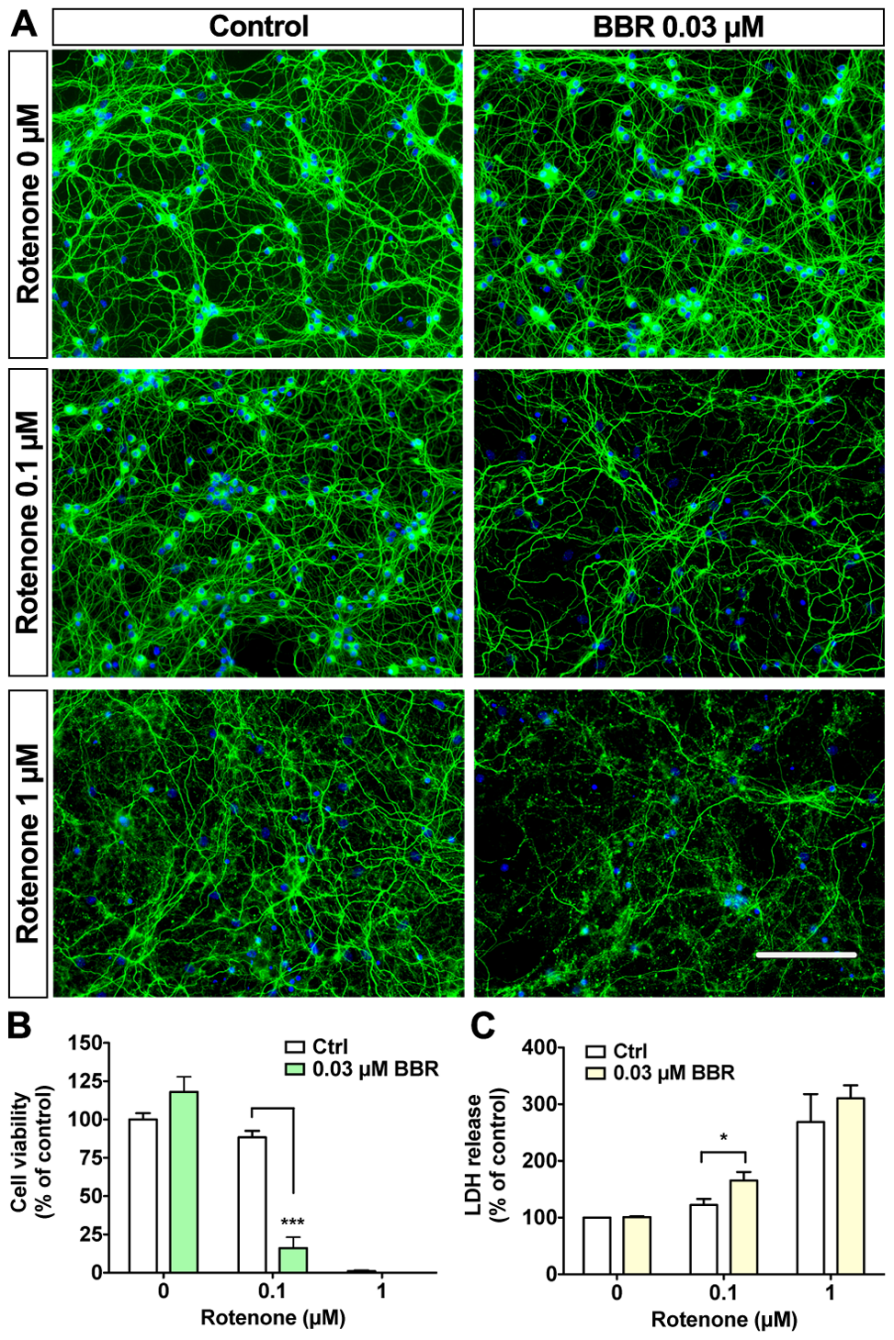
Berberine sensitizes neurons to rotenone-induced injury. Pretreatment with 30 nM BBR is sufficient to sensitize CGN to rotenone toxicity. (A) Representative images of CGN pretreated for 18 hours before the addition of 0.1 or 1 µM rotenone for 6 hours. (B) Quantification of CGN cell viabilities shown in A. (C) LDH release assay shows exacerbation of toxicity by BBR pretreatment. For A, green is β-tubulin III, blue is Hoechst; the scale bar represents 100 µm. For panels B and C, n = 3. For B and C, *  = p<0.05, ***  = p<0.001.

The interlinked roles of NMDA receptors and mitochondria are central to excitotoxic injuries resulting from glutamate exposure and perturbations in the respiratory chain [40], [41], [48]. Mitochondrial complex I is a common target of pesticides, including rotenone, which is a potent mitochondrial complex I inhibitor with an IC_50_ of ≈2 µM [49], [50]. Moreover, BBR is suggested to directly interfere with mitochondrial complex I [11]. Rotenone is particularly toxic to dopaminergic neurons and is commonly used in Parkinson's disease models [51]. In CGN, rotenone causes acute toxicity within an hour at 5 µM [52] and 10–20 nM levels severely restrict the oxidative respiration rate [40]. BBR has properties similar to these known mitochondria-targeting toxins, as discussed by Shin et al. [19]. We investigated the effects of BBR pretreatment on rotenone toxicity. Our data indicated that 1 µM rotenone caused rapid toxicity within 6 hours, whereas 0.1 µM rotenone did not significantly affect CGN gross morphology and cell viability following a 6-hour treatment (Figure 6A). However, 30 nM BBR pretreatment for 18 hours dramatically sensitized CGN to rotenone treatment, decreasing cell viability from 88.3±4.3% down to 16.0±7.2% in the presence of 0.1 µM rotenone as assessed by nuclear morphology (Figure 6B) and LDH release assay (Figure 6C). Interestingly, the neuronal network remained relatively intact following rotenone treatment, suggesting a more prominent effect on the neuronal soma than the neuritic network. These data indicate the central role of NMDA receptors and mitochondria in BBR neurotoxicity and that subtoxic levels of BBR can sensitize neurons to excitotoxicity and mitochondrial toxins [53].

## Discussion

In this study, we explored the concentration-dependent effects of BBR, a widely used nutraceutical, on the viability of cultured primary neurons. In contrast to the general view of BBR as a neuroprotective nutraceutical, we report that BBR causes neurotoxic effects on cultured cerebellar granule neurons (CGN) and hippocampal neurons (HCN). Low micromolar BBR concentrations induced rapid caspase-independent cell death, which was associated with functional and morphological alterations of mitochondria. Neurotoxicity of BBR was completely blocked by the NMDA receptor antagonists memantine and MK-801, with partial protection achieved with the mitochondrial PTP inhibitor cyclosporine A. Additionally, BBR pretreatment sensitized neurons to glutamate and rotenone injury at similar concentrations previously associated with neuroprotection [16]. Our study raises concerns of acute BBR neurotoxicity due to its prominent effects on mitochondria and NMDA receptors, especially when applied at micromolar concentrations close to the levels suggested to be attainable by oral dosing.

BBR is generally well tolerated, as evidenced by its long history of traditional use and several controlled clinical trials in metabolic disorders [5], although use restrictions have been enforced in certain countries, such as Singapore [54], [55]. Current literature supports the use of BBR against various indications, ranging from antibiotic and anti-diabetes to anti-cancer and neuroprotection [20]. However, the range of concentrations, treatment times, cell lines, animal models and application methods vary greatly between studies, making direct comparison between published reports difficult. Pharmacological studies on animals and humans suggest that BBR has poor bioavailability. However, while plasma concentrations only reach low nanomolar range, BBR can accumulate in numerous organs, including the brain, reaching low micromolar concentrations [13]. Current attempts to increase the bioavailability of BBR may enhance efficacy when used in metabolic indications, but our study highlights the importance of a thorough safety assessment of BBR in the nervous system. This would be especially important considering the potential long-term use of BBR in the aging population suffering from metabolic diseases, which themselves may increase the risk of ischemic injury and neurodegenerative diseases such as Alzheimer's disease [56].

Our findings support recent evidence that BBR may exacerbate neurotoxicity in dual-hit conditions, as proposed by Myung Koo Lee et al. in Parkinson's disease models [19], [57]. Contrary to this view, BBR has been proposed to act as a neuroprotective nutraceutical against various apoptotic insults arising from neurodegenerative conditions such as ischemia and Alzheimer's disease, based on both animal models and several cell lines, including SH-SY5Y, NSC34, and PC12 cells [13], [16], [18], [58]–[61]. Based on previous literature, however, the effects of BBR on cell viability are highly variable. For example, at low nanomolar concentrations (0.1–10 nM), BBR protects PC12 cells against apoptosis by decreasing caspase activation and ROS generation [27]. However, PC12 cells differ significantly from the primary neurons used in this study. Neurons are generally more sensitive to changes in energy metabolism, excitation and ionic balance in comparison to cell lines [40]. Moreover, PC12 cells lack functional NMDA receptors [62]. These crucial inherent cell physiological differences could explain the robust BBR toxicity observed in primary neurons, leaving many other cell types unaffected at low micromolar concentrations. Whether the protective effects of BBR in PC12 and other cell lines could also be seen with micromolar BBR remains to be seen.

Pro-apoptotic and anti-cancer effects are attributed to high micromolar concentrations of BBR that efficiently inhibit tumor cell proliferation, DNA synthesis, and induce cell cycle arrest [27]. There is evidence for both caspase-dependent and -independent pro-apoptotic roles for BBR [32], [33]. We describe BBR-mediated neurotoxicity as a rapid, caspase-independent process. With an IC_50_ of roughly 3 µM, BBR caused rapid nuclear condensation and fragmentation of neuronal dendrites and axons within 4–6 hours. The radical drop in ATP levels and reduced mitochondrial metabolic activity after micromolar BBR application indicates that BBR causes a severe energy depletion in neurons, resulting in caspase-independent cell death, as ATP would be needed for programmed apoptotic cell death [63]. This is further supported by the insensitivity of BBR-treated cells to pan-caspase inhibitor z-VAD-FMK and the absence of c-Jun phosphorylation and cleavage of caspase-3, a major executioner caspase.

Mitochondrial targeting is a property of BBR already noted in the 1970s [64], [65]. The crucial involvement of mitochondria is evident from the rapid alterations of mitochondrial function and morphology following BBR addition. BBR, and its derivative dihydroberberine, have been shown to specifically target the mitochondrial complex I [11]. Recently, Pereira et al. characterized the effects of BBR on melanoma cell mitochondria as well as isolated mitochondrial fractions [22], [35]. They demonstrated that BBR can accumulate in mitochondria causing mitochondrial depolarization and fragmentation, mitochondrial PTP induction, increased oxidative stress, decreased cellular ATP content, and cell cycle arrest [35]. Our results from primary neurons support these findings. While these effects may be desirable for antitumor agents, they may also cause toxicity in neurons, which are sensitive to metabolic disturbances and thus particularly sensitive to mitochondrial dysfunction [40], [41], [63], [66]. Our current results suggest that mitochondria are centrally involved in the toxic effects of BBR in neurons.

In addition to mitochondrial effects, BBR has been reported to modulate ionic currents, particularly via potassium channels [67]. At concentrations similar to the IC_50_ of ≈3 µM for CGN viability, BBR inhibits delayed rectifier currents and HERG channels [15], [25], [68]. At higher concentrations, BBR also inhibits a variety of potassium currents, including inward and outward rectifier, voltage sensitive, and K^+^ channel currents [25], [67]–[69]. The IC_50_ for outward rectifier current is around 10 µM. Moreover, BBR blocks ATP-sensitive potassium (K_ATP_) channels with an IC_50_ of 13 µM, which may cause further depolarization [69]. Interestingly, NMDA receptor composition can be modulated by BBR [70]. We found that the neurotoxic effects of BBR can be completely blocked by inhibition of NMDA receptors, suggesting that modulation of cellular excitability by BBR significantly contributes to its neurotoxic mechanisms.

Our study confirms previous reports on the neurotoxicity of BBR [19], [57] and suggests a mechanistic basis to understand how BBR could enhance neurodegenerative processes. These findings raise concerns over the CNS safety profile of BBR, particularly when used in the long-term in the aging population, in patients at risk of silent strokes or ischemic episodes [71], or in people at risk of chronic systemic pesticide exposure [50], [72]. Importantly, our results also suggest that memantine, a clinically available NMDA receptor antagonist, may be used to protect neurons against BBR toxicity. Widely available nutraceuticals and dietary supplements have gained considerable interest due to their potential health effects and presumed safety. However, more attention should be paid to both regulatory and research needs in this field.
